# Association of BMI, Physical Activity with Academic Performance among Female Students of Health Colleges of King Khalid University, Saudi Arabia

**DOI:** 10.3390/ijerph182010912

**Published:** 2021-10-17

**Authors:** Amani Alhazmi, Farah Aziz, Manal Mohammed Hawash

**Affiliations:** 1 Department of Public Health, College of Applied Medical Sciences, King Khalid University, Khamis Mishit 61421, Saudi Arabia; amanialhazmi@kku.edu.sa (A.A.); mamomohammed@kku.edu.sa (M.M.H.); 2 Department of Basic Medical Sciences, College of Applied Medical Sciences, King Khalid University, Khamis Mishit 61421, Saudi Arabia

**Keywords:** BMI, academic performance, physical activity, lifestyle, health college students

## Abstract

Body mass index (BMI) is an indicator to detect weight category and known to influence the academic achievements of students. The present study assesses the association of BMI, physical activity with academic performance among undergraduate female students of health colleges, King Khalid University (KKU). Responses from 379 participants were analyzed in the study. Data collection was done by administering an online questionnaire using the university website portal. The questionnaire consists of two sections: the first section includes demographic information along with cumulative Grade point average (GPA) and another section rated student’s physical activity. A high frequency of respondents (53.6%) ranged within the normal BMI category. For academic performance, the majority (79.9%) of students reported high GPA scores with a mean of 4.28 ± 0.41. Upon correlation, academic performance was found negatively correlated with BMI at 0.0001 level of significance, and insignificantly correlated with physical activity. The present study observed that more than half of the respondents had normal BMI. An inverse relationship was observed between BMI and academic performance, showing participants within the normal BMI category achieved significantly higher GPA. In contrast, physical activity directly influenced the academic performance of the subjects. Therefore, the study suggests to enact counselling centers, health clubs in universities urging the students to adopt regular exercise and healthy lifestyle which could prepare them in achieving future endeavors.

## 1. Introduction

The prediction and explanation of academic performance and the investigation of the factors relating to the academic success of students are issues of utmost importance in higher education. The college period could be a stressful phase as a result of significant changes at educational level and social level. Undergraduates portray diverse ways of lifestyle including physical activity and dietary patterns [1]. The utmost imperative lifestyle changes were eating more snacks between meals, spending extensive time using smartphones and computer for entertainment instead of exercise participation. Therefore, obesity becomes a more prevalent serious problem among university students which certainly has negative consequences on the health outcomes of undergraduates [2]. Numerous studies with significant frequencies reported the obesity among college students [3,4,5].

Obesity occurs as a result of extreme escalation in the body’s fatty mass percentage to the muscle mass when the proportion of height to weight surpasses the optimum level. Body mass index (BMI) is globally applied as a screening index to evaluate levels of the bodies’ morphological development and nutritional status, which is closely related to fat contents, long-term health effects, and disease risks. Thus, BMI is a considerable indicator to detect weight problems [6]. BMI has been widely used to discover the relationship between weight problems including obesity and academic achievement. Wehigaldeniya et al. showed that obese university students are more susceptible to psychological problems such as anxiety, depression and more likely to be socially isolated which in turn influences their academic attainment [7]. Cognitive ability and memory functions are well-established predictors of academic performance [1]. Numerous studies have been linked obesity to poorer adult cognitive functioning, such as diminished visual memory performance, greater delay discounting, neurostructural deficits evident in the orbitofrontal cortices, and impairments in verbal memory, attention span, and decision making [8,9,10,11].

A decline in adult physical activity at university stage is considered as a vital risk factor for developing overweight and obesity [12]. Previous studies claimed that exercise helps get better academic performance by increased secretion of serotonin, improved cerebral circulation, hormone levels changes, and increased self-esteem [13,14]. Other researchers have shown a positive association between the level of physical activity and intellectual functioning, particularly the working memory capacity, reaction time, and acuity [15,16].

Subsequently, university students are an important segment of the population, and their health relates to the upliftment of the society. Despite both BMI and physical activity have been shown to influence academic attainment, data for college-age students are limited and often indistinct. Further, there is inconsistent evidence regarding the nature and magnitude of the association between BMI and academic achievements among university students. Due to availability of few studies regarding the effect of physical activity, BMI on university students’ academic outcomes were investigated, thus, the current study is sought to examine the association of BMI, physical activity and academic performance among female students of health colleges of King Khalid University.

## 2. Materials and Methods

### 2.1. Subjects

This was a cross-sectional study and the convenient sampling method was used. Sample size was calculated using Cochran’s Formula. A total of 387 responses were recorded, out of which 8 incomplete questionnaires were removed, resulting in 379 responses for analysis. All participants were undergraduate female students aged between 18–30 years and belong to different health colleges; College of applied medical sciences (Public Health, Nursing, Medical Laboratory Science, Physiotherapy), Medicine and Surgery, Dental and Oral surgery, Diagnostic Radiology, Pharmacy and Emergency medicine of KKU, Saudi Arabia. Females of Saudi ethnicity, from all health colleges of KKU from level four to nine who agreed to participate voluntarily in the study were included.

### 2.2. Data Collection

Data collection was done by a self-administered online questionnaire, disseminated using university website portal. Data were collected for a period of one month, from 1 June to 30 June 2021. The questionnaire consisted of two sections: the first section encompasses anthropometric and demographic information, such as age, weight, height, BMI, marital status, department, level of study, overall cumulative GPA with standard 5.0 scale, and additional question to ensure they are free of chronic disease or not. The second section assessed the students’ physical activity pattern, and non-physical activities.

BMI was calculated by Cole’s formula and used to check the weight category. According to the National Institute of Health, adults were classified depending upon their BMI as normal (BMI = 18.5–24.9 kg/m^2^), overweight (BMI = 25–29.9 kg/m^2^), or obese (BMI ≥ 30 kg/m^2^).

GPA was assessed as per National center for assessment in Higher Education (NCAHE) and National Commission for Academic Accreditation and Assessment (NCAAA): Grade 95–100 = 5.0 = A+ Excellent, Grade 90 to less than 95 = 4.75 = A Excellent, Grade 85 to less than 90 = 4.5 = B+ Very Good, Grade 80 to less than 85 = 4.0 = B Very Good, Grade 75 to less than 80 = 3.5 = C+ Good, Grade 70 to less than 75 = 3.0 = C Good, Grade 65 to less than 70 =2.5 = D+ Pass, Grade 60 to less than 75 = 2.0 = D pass, Grade Less than 60 = 1.0 = F Fail [17].

### 2.3. Statistical Analysis

Statistical analysis was performed using SPSS version 25. Descriptive statistics were used to describe different quantitative and qualitative variables. Pearson coefficient is used to correlate between two normally distributed quantitative variables. Independent-sample t-test is used to determine whether there are any statistically significant differences between the means of two independent groups. One-way analysis of variance (ANOVA) was used to find the difference between different variables. The Cohen effect size convention was used to measure strength of the relationship between variables in a population at ≤0.05 and ≤0.01 level of significance [18].

### 2.4. Ethical Considerations

The ethical Committee of King Khalid University approved this study. The studied subjects were informed about the purpose of study and they could withdraw at any time.

## 3. Results

Table 1 shows the characteristics of study participants.

Table 2 shows that a high frequency of the respondents comes within the normal BMI range. Around 20.1% of the study subjects were overweight followed by underweight and a minimum were in the obese category. The coefficient of variation among different BMI categories was observed to be 37.2%.

Table 3 illustrates the majority of the participants attained a high academic performance level with a coefficient of variation of 22.7%.

Table 4 depicts the significant negative relationship among overall academic performance level and total BMI at 0.000 level of significance. A positive but insignificant relation was seen among the overall level of academic performance and physical activity pattern domains.

Table 5 shows the difference between BMI scores of students with high and low academic performance. In addition, it was observed that the study participants with high GPA had a normal BMI compared to students who attained low GPA. The effect size of total BMI scores on overall academic performance was high according to the Cohen effect size convention (1988) [18].

Table 6 displays respondents without any chronic diseases showed significantly higher overall mean of academic performance when compared to participants with chronic illness (*p* = 0.000). In addition, it denotes students with chronic illness have higher BMI scores than students without any chronic illness (*p* = 0.026). The frequency of 38.8% of physically active respondents showed a significantly higher overall mean of academic performance than 61.2% of physically inactive students. Furthermore, this table reveals that out of those who practiced physical activity, half practiced for 1–2 h weekly, as well as the studied subjects who exercise more than 4 h weekly have significantly higher overall mean of academic performance than the participants who either did less than 4 h or not exercise at all (*p* = 0.023). Moreover, there is no statistically significant difference between total mean BMI scores and all physical activity domains of the studied undergraduates.

## 4. Discussion

Academic learning is a crucial phase marked by physiological maturity and health-related behavioral transitions. Individual health status and quality of life should be reliant on human practices and lifestyle [19]. Healthy behavior is imperative and considered one of the most receptive predictors that determine students’ learning achievement [20]. Meanwhile, academic life is characterized by impulsive behaviors, resulting in changes in nutritional and physical activity pattern due to peer groups and media power. Consequently, university students are a distinct group that is vulnerable to many disorders related to BMI including obesity, overweight, and underweight problems [21]. The present study was conducted due to availability of a few studies regarding the effect of physical activity, BMI on university students’ academic outcomes.

BMI might be identified as an incentive that prompts people to apply certain approaches for improving their pattern of life. Body image and its connection to self-concept are very significant, particularly among younger women. Thereby, they usually maintain their weight within the normal limit [22]. The present study confirmed this hypothesis and showed that the overall mean BMI of the studied participants was within the normal range (23.98 ± 0.74 kg/m^2^). Our results are consistent with those of Al-Momani M [23] who revealed that the overall mean BMI of the respondents was 24.9 ± 6.4 kg/m^2^ (range, 14.37 to 36.43 kg/m^2^). In addition, this study showed that normal BMI was prevalent in more than half of the study participants. This was congruent with Ikujenlola et al. [24] as well as Alazayani et al. [25] who reported that the BMI of the undergraduates was within the normal level.

Obesity is a serious public health disorder, which has numerous implications on learning achievement, particularly when it becomes more prevalent in the Arab Gulf region [21,25,26]. The present study showed that 20.1% and 12.9% of the undergraduates were overweight and obese, respectively. These findings were supported by Alazayani et al. and others [25,27] who reported that nearly one-fifth of the medical students were overweight and more than one-tenth were obese. Similarly, Sirang et al. [28] revealed that overweight was prevalent among 21.3% of the subjects. Contrarily, various researchers observed the overweight and obese respondents with the least frequency [23,26].

Body-image dissatisfaction among female college students might be the primary antecedent for the growing behavior of unhealthy weight loss, which may be detrimental to young women’s physical wellbeing [22,23]. Our results reported that 13.5% of the studied subjects were underweight. This could be attributed to the culture which is considered a powerful incentive to the popular misconception that a slimmer body makes a woman more attractive, resulting in unhealthy behavior that regulates weight, including over-exercise, and eating disorders. This is in line with numerous studies who reported noteworthy frequency of underweight female students [23,25,26,28,29].

Maintaining healthy behaviors, and the quality of learning processes are considered vital factors for the students’ educational outcomes [19,20]. Hereby, we revealed that the majority of the undergraduates showed high-level academic performance. This could be related to the fact that diligence, enthusiasm, commitment, and time-management skills are positive correlates for academic achievement, which is more widely shown, particularly in the undergraduates of medical and other health-related colleges. These results are consistent with those of Muhammad et al. [26] and Al-Momani M. [23]. However, these conclusions are not in agreement with those of Aleidi et al. [30] who reported that nearly one-half of college students attained good GPA.

In the association between the body mass index of learners and their educational achievements, our study suggested and predicted that an individual could academically do well according to their BMI, and vice versa. The current study findings proved this hypothesis and reported that the undergraduates with high GPA had significantly normal BMI while the studied undergraduates who attained low GPA were overweight or obese BMI. A statistically significant negative association were also found between the overall BMI and academic performance of the study subjects. These findings were in line with Jinbo et al. [31] and Muhammad et al. [26] who revealed that there is a weak and negative correlation between BMI and the academic performance of female participants. Similarly, Anderson et al. [32] demonstrated a significant negative correlation between BMI or obesity and academic performance. An observation by Bahammam et al. [33] showed that a lower obesity prevalence among excellent students rather than average ones. In addition, the present results are supported by the findings of Suraya et al. [34], a study of Saudi Arabia. Contrarily, a study carried out in Siri Lanka [7] stated that BMI is not related to academic performance. However, the present results were congruent with those of Aleidi et al. [30] who reported that students who were obese or overweight, and had higher BMI were significantly correlated with a lower GPA compared to the students with excellent or very good GPA. Further, this study concluded that the effect size of total BMI scores of the studied participants on overall academic performance was high. These were congruent with other studies [35,36,37]. In contrast, several studies contradicted these results and reported that there was a low effect size of BMI on academic performance [38,39,40].

Regular physical exercise has an active role in sustaining healthy lifestyle behaviors. It is also having a beneficial positive impact on intellectual functioning [19,20]. The current study proved this hypothesis and claimed that more than one-third of the participants who were active have a significantly higher overall mean of academic performance than the studied participants who were inactive. As well, the studied subjects who did exercise more than four hours weekly have a significantly higher overall mean academic performance than the participants who either did less than four hours weekly or not exercise at all. These could be related to numerous explanations. Firstly, regular physical activity is associated with weight loss and better overall health, especially cognitive capabilities. Secondly, because the majority of participants have not had any chronic illness, they did all academic activities, which require physical health such as attending classes that further help them in their academic studies. Thirdly, more than one-half of the participants had normal weight which aids them to do a higher level of physical exercise than overweight students which in turn enhances their academic performance. These findings were supported by Hillman et al. [41] who concluded that consistent engagement in physical exercise is connected to improving mental and cognitive functioning, and thus significantly affecting the student’s educational outcomes. Similarly, Muhammad et al. [26] claimed that there was a medium significant association between physical health and academic achievement among Nigerian students. This was also in coherence with the findings of Cid and Muñoz [42] that indicate a positive correlation between physical exercise and academic achievement. Comparably, Burke [43] found that female student nurses who were more inactive and have lower fitness were reported lower academic levels. Surprisingly, this study findings found that there is not a significant correlation between the total mean BMI scores and physical activity of the studied undergraduates. In contrast, Muhammad et al. [26], Alazayani et al. [25], and Atare et al. [35] proved a statistically positive significant difference between BMI and physical activity.

An individual’s health status is the most significant indicator for being actively participating in normal daily life activities together with academic activities. Chronic illness could impact every aspect of a student’s life causing significant educational challenges, which ultimately affect educational outcomes [19,22,29]. This research confirmed this fact and revealed that female medical undergraduates who reported that they have not any chronic illnesses had a statistically significantly higher overall mean of academic performance than the studied participants who have one and more chronic illnesses. These study findings were supported by Al Ghamdi S. [22] and El Ansari [44] who reported that health status, health behaviors, and health complaints were selectively associated with indicators of student educational achievement. However, these findings were incongruent with those of Aleidi et al. [30]. They reported that there is a correlation between the majority (87.0%) of the studied students with no medical conditions and academic performance with no statistically significant difference. Zaher et al. [45] also contradicted these study results and documented that chronic illnesses among medical students have no significant impact on their academic performance.

This study has some limitations such as the students’ GPA was assessed by self-reporting based on the students’ memories which increases the likelihood of human error. This risk of human error could be eliminated by receiving an official GPA report from the administrative unit.

## 5. Conclusions

The current study found that more than one-half of the participants had a normal BMI, and one-fifth of them were overweight. More than one-tenth of students had either obesity or underweight problems. The overall academic performance was significantly negatively correlated with BMI among the students. The participants with normal BMI also significantly attained higher GPA, however, those with low GPA were significantly correlated with high BMI in different health specialties of King Khaled University, KSA. Further, the study has shown that physical activity is significantly positively affecting the undergraduates’ academic performance. Moreover, this research claimed that medical undergraduates without any chronic diseases have a statistically significantly higher overall mean of BMI and academic performance. Therefore, this study suggested that universities should take an active role to establish counseling centers, and health clubs for the students on the university campus to promote the students’ wellbeing, and healthy lifestyle behaviors, such as regular exercise and weight control. Future research is required to examine whether or not the associations that were observed between academic performance and BMI, and physical activity in this cross-sectional study represent causal relationships.

## Figures and Tables

**Table 1:** Demographic and general information of the study participants (n = 379).

Variable	Categories	No	%
Age	18–23	375	98.9
	≥24	4	1.1
Marital status	Unmarried	350	92.4
	Married	29	7.6
Height (Mean ± SD)	158.17 ± 10.99	379	100
Weight (Mean ± SD)	60.02 ± 15.35	379	100
BMI (Mean ± SD)	23.91 ± 6.83	379	100
College/Department	Nursing	45	11.9
	Emergency medicine	3	0.8
	Public health	48	12.7
	Radiology	26	6.9
	Pharmacy	116	30.6
	Dentistry	49	12.9
	Medicine	35	9.2
	Physiotherapy	34	9.0
	Medical Laboratories	23	6.0
Study Level	Fourth	109	28.8
	Fifth	44	11.6
	Sixth	44	11.6
	Seventh	43	11.3
	Eighth	58	15.3
	Ninth	17	4.5
	Tenth	64	16.9
Do you suffer from any chronic disease?	Yes	31	8.2
	No	348	91.8

SD—Standard deviation, No—Number.

**Table 2:** Body mass index (BMI) classification among the studied participants (n = 379).

BMI Classification	Range	No	%	(Mean ± SD)	Total Mean of BMI Scores (Mean ± SD)	Coefficientof Variation
Underweight	<18.5	51	13.5	17.34 ± 0.88	23.91 ± 6.83	37.2%
Normal weight	18.5–24.9	203	53.6	21.54 ± 1.91		
Overweight	25–29.9	76	20.1	27.03 ± 1.45		
Obesity	≥30	49	12.9	35.78 ± 10.88		

**Table 3:** Academic performance level among the study participants (n = 379).

Academic Performance Level	Range	No	%	(Mean ± SD)	Overall Mean of Academic Performance (Mean ± SD)	Coefficient of Variation
Low	1.4–2.5	76	20.1	2.79 ± 0.53	3.98 ± 0.74	22.7%
High	2.6–5	303	79.9	4.28 ± 0.41		

**Table 4:** Correlation among the overall academic performance level, total BMI and physical activity pattern of the studied subjects (n = 379).

Variables	Overall Academic Performance r-Value	*p*-Value
Total Body mass index (BMI)	−0.391	0.000 **
Physical activity pattern	0.010	0.876
How many hours do you perform exercise?	0.048	0.367
What do you prefer to do in your spare time rather than exercise?	0.047	0.308
How many hours do you spend on non-physical activities?	0.022	0.721

** < 0.01 level of significance.

**Table 5:** Mean ± SD and differences between total BMI scores, and academic performance levels of the study participants (n = 379).

	High Academic Performance (n = 303)	Low Academic Performance (n = 76)	Significant Difference		Effect Size
	Mean ± SD	Mean ± SD	t	*p*-Value	(η^2^)-Value
BMI	22.48 ± 1.99	29.61 ± 11.37	8.956	0.000 **	−0.175

Normal BMI: 18.5–24.9 Overweight BMI: 25–29.9 Obesity BMI: ≥30, ** *p* < 0.01 level of significance.

**Table 6:** Mean and significant differences among total BMI scores, overall academic performance level, clinical and physical data, and non-physical activity pattern of the studied participants (n = 379).

Variable	Categories	No	%	Overall Level of Academic Performance	Total BMI Scores
				Mean ± SD	Mean ± SD
Presence of chronic disease	Yes	31	8.2	3.50 ± 0.88	26.53 ± 9.20
	No	348	91.8	4.03 ± 0.71	23.68 ± 6.54
t-Value				3.888	2.235
*p*-value				0.000 **	0.026 *
Physical activity pattern	Extremely inactive	39	10.3	3.89 ± 0.78	24.41 ± 6.72
	Inactive	193	50.9	3.89 ± 0.79	24.69 ± 6.57
	Active	130	34.3	4.08 ± 0.67	23.31 ± 7.11
	Extremely active	17	4.5	3.99 ± 0.85	23.75 ± 5.59
F-Value				3.106	1.120
*p*-value				0.027 *	0.341
How many hours did you do exercise?	1–2 h weekly	200	52.8	3.89 ± 0.79	23.86 ± 7.36
	3–4 h weekly	123	32.4	4.00 ± 0.67	23.17 ± 5.67
	>4 h weekly	17	4.5	4.79 ± 0.85	23.02 ± 4.09
	No exercising	39	10.3	3.39 ± 0.78	24.61 ± 7.10
F-Value				3.219	0.804
*p*-value				0.023 *	0.492
What did you do in your spare time?	Shopping	43	11.3	3.86 ± 0.69	22.56 ± 4.85
	Social media	249	65.7	3.99 ± 0.74	24.25 ± 6.99
	Reading	58	15.3	3.99 ± 0.69	23.66 ± 8.46
	Watching TV	29	7.7	4.05 ± 0.92	23.57 ± 3.33
F-Value				0.457	0.816
*p*-Value				0.713	0.486
How many hours did you spend on non-physical activities?	1–2 h daily	45	11.9	4.03 ± 0.73	23.55 ± 5.50
	3–4 h daily	141	37.2	3.96 ± 0.76	24.30 ± 6.43
	5–6 h daily	193	50.9	3.99 ± 0.73	23.72 ± 7.38
F-Value				0.126	0.363
*p*-Value				0.881	0.696

* *p* < 0.05; ** *p* < 0.01 level of significance.

## Data Availability

Not applicable.
